# Role of Oxidative Stress in Obese and Nonobese PCOS Patients

**DOI:** 10.1155/2022/4579831

**Published:** 2022-02-09

**Authors:** Kazım Uçkan, Halit Demir, Kasım Turan, Eren Sarıkaya, Canan Demir

**Affiliations:** ^1^Van Training and Research Hospital, Department of Obstetrics and Gynecology, Van, Turkey; ^2^Van Yuzuncu Yil University, Department of Biochemistry, Van, Turkey; ^3^Clinic of Obstetrics and Gynecology Dr. KasımTuran, Van, Turkey; ^4^Van Yuzuncu Yil University, Vocational School of Health Services, Van, Turkey

## Abstract

**Objective:**

The aim of this study was to evaluate the relationship between the oxidant-antioxidant status, endothelial dysfunction, lipid metabolism, and metabolic syndrome risk in women with polycystic ovary syndrome (PCOS).

**Materials and Methods:**

Forty-five obese (BMI >30 kg/m2) woman diagnosed with PCOS in the study, forty-five nonobese (BMI <30 kg/m^2^) PCOS diagnosis working groups, and forty-nine healthy control groups were created with patients. Serum malondialdehyde (MDA) levels with antioxidant activities, such as SOD, GSH, GPx, and CAT activities, were measured by spectrophotometry.

**Results:**

There was a statistically significant difference in the mean serum MDA level in the obese PCOS group compared to the nonobese group and the control group (*p* < 0.001). When the antioxidant parameters, such as SOD, GPx, GSH, and CAT, were compared with the healthy control group, nonobese, and obese PCOS groups, the difference between the groups was statistically significant (*p* < 0.001). A positive correlation was observed between MDA and BMI, triglyceride, LDL, SBP, DBP, and HOMA-IR in the PCOS patient group.

**Conclusion:**

Oxidative stress and decreased antioxidant parameters in PCOS patients were correlated with hyperinsulinemia, hypertension, and dyslipidemia findings, and we think that this oxidative stress condition may contribute to metabolic syndrome and cardiovascular diseases in PCOS patients.

## 1. Introduction

Polycystic ovary syndrome (PCOS) is a common endocrine disorder that affects 6% to 20% of women at reproductive age [1]. Usually, women with PCOS have hyperandrogenism and hirsutism, oligo or amenorrhea, and anovulation. Although there is a long history of study on PCOS, its etiology is still unknown. Today, the role of inflammatory mechanisms, endothelial damage, oxidative stress, and genetic mechanisms are explained [2, 3].

It has been reported that women with polycystic ovary syndrome have abnormalities in estrogen and androgen metabolism. In one study, it has been determined that PCOS may be caused by abnormal function of the hypothalamic-pituitary-ovarian (HPO axis). In mammalian ovaries, LH has been found to induce androgen biosynthesis by the theca interna cells. However, FSH has been found to stimulate aromatase activity by the granulosa cells. The coordinated action of these two cells and pituitary hormones has been found to form the basis of the bicellular, bigonadotropin hypothesis for estrogen biosynthesis [4].

The characteristic features of PCOS include LH hypersecretion both basally and in response to GnRH. Therefore, LH hypersecretion can produce androgen in excess. Thus, the primary abnormalities in classic PCOS occur. LH/GnRH pulses cause hyperandrogenemia and impaired follicular maturation in PCOS patients. This renders it insensitive to estrogen/progesterone inhibition of hypothalamic GnRH formation. As a result of this insensitivity, perimenarchal abnormalities in hyperandrogenemic PCOS women may be a potential mechanism [5].

Cells have an efficient antioxidant defence system, mainly composed of enzymes, such as superoxide dismutase (SOD) and glutathione peroxidase (GSH-Px). They can reactivate oxygen species (ROS) produced by cellular metabolism and make ROS level stable under physiological conditions [6].

The rich sources of polyunsaturated fatty acids (PUFA) easily attack the cell membranes under the influence of oxidizing radicals. This process is known as lipid peroxidation, and malondialdehyde (MDA) is one of the important end products of this process. It correlates with the degree of lipid peroxidation and is, therefore, one of the most common biomarkers used to assess oxidant status [7]. Insulin resistance, hyperandrogenism, dyslipidemia, and PCOS-related obesity probably increase MDA levels and also reduce antioxidant enzyme levels [8].

Oxidative stress is called an imbalance between the oxidants and antioxidants and the formation of excessive reactive oxygen species (ROS). Numerous studies have shown that markers in the oxidative circulation are greater than normal in patients with PCOS and that oxidative stress is important in the pathogenesis of PCOS [9, 10]. It is thought that relative insulin resistance results in chronic hyperinsulinemia, which gives rise to abnormal ovarian androgen metabolism, impaired follicle growth, and altered gonadotrophin response [11]. Reactive oxygen species production and oxidative stress have been linked to insulin resistance. Oxidative stress impairs glucose uptake in the muscle and adipose tissue and reduces insulin secretion from the pancreatic *β* cells [12]. In addition, because of hyperinsulinemia in PCOS patients, nitric oxide (NO) secretion is prevented from vascular endothelium. As a result, the membrane fluid decreases, which leads to an increase in intracellular calcium levels, causing endothelial dysfunction. Thus, endothelial dysfunction, an early symptom of atherosclerosis, may occur [13]. ROS production increases in response to PCOS, and various features of PCOS, including abdominal adiposity, insulin resistance, obesity, and androgen excess, have the potential to improve oxidative stress in these patients [14]. Atherosclerotic heart disease (AHD) is associated with an imbalance between the oxidant levels and the cellular antioxidant defense mechanism. Increased oxidative stress may facilitate the development of AHD [15].

The purpose of this study is to explain the relationship between the antioxidants and oxidative stress in obese and nonobese polycystic ovary syndrome (PCOS).

## 2. Methods

PCOS is the most common cause of anovulatory infertility that affects the reproductive, endocrine, and metabolic systems. Inclusion criteria for the study population were ages varying between 18 and 30 years in this study, and PCOS was diagnosed using the Rotterdam criteria [1]. According to these diagnostic criteria, hyperandrogenic symptoms, such as hirsutism, acne, and androgenic alopecia, the presence of oligomenorrhea (where menstrual cycles occur more than 35 days apart), amenorrhea (wherein menstrual cycles are at least 6 months apart), at least 12 follicles measuring 2 mm to 9 mm in diameter, and/or an ovarian volume greater than 10 cm^3^ per ovary indicated the existence of polycystic ovaries. Additionally, the presence of hirsutism was evaluated using the Ferriman–Gallwey scoring system [16]. Body mass index (BMI) was calculated by dividing the body weight (kg). 45 patients with BMI <30 kg/m^2^ were included in the nonobese group and 45 patients with BMI> 30 kg/m^2^ were included in the obese group. The waist circumference (WC) in centimeters (cm) was obtained with a nonstretchable measuring tape placed at the approximate midpoint between the lower border of the last palpable rib and the top of the iliac crest. The hip circumference (HC) in cm was also obtained with the nonstretchable measuring tape placed around the widest portion of the buttocks, using the greater trochanter of the femur as a landmark. The waist-hip ratio (WC : HC) was then obtained by dividing WC by HC. The WHO recognizes a WC : HC ratio of greater than 0.85 as abdominal obesity in women [17]. The homeostasis model assessment of insulin resistance (HOMA-IR) was calculated using the following formula: (fasting insulin (*μ*IU/mL) × fasting glucose (mmol/L))/22.5. Subjects were considered to have IR when the HOMA-IR index was ≥2.6 × 10^−6^ mol × U/L2 [18].

Exclusion criteria for the study: diabetes mellitus, chronic hypertension, cardiovascular patients with thyroid dysfunction, endometriosis, and patients taking lipid-lowering or insulin-sensitizing drugs were not included in the study. The patient and control groups were of indistinguishable socioeconomic status. All volunteer patients were informed about the study conducted according to the Helsinki Declaration as received in 2000. The study was approved by the Local Ethics Committee.

### 2.1. Blood Collection

Blood samples were collected from the Gynecology and Obstetrics Clinic Van Regional Training and Research Hospital. Blood samples were collected into biochemistry blood tubes. Serums were separated by centrifugation at 5.000 rpm for 10 minutes and stored at −85°C until use.

### 2.2. Measurement of Serum Lipid Peroxidation

Serum malondialdehyde level (MDA) was measured spectrophotometrically by UV-VIS spectrophotometer by the thiobarbituric acid method. The method was performed as follows: 200 *µ*l of serum samples were taken into each tube. 800 *µ*l phosphate buffer, 25 *µ*l BHT solution, and 500 *µ*l 30% TCA were added. The tubes were mixed in the vortex, capped, and kept in an ice bath for 2 hours. The tubes were readied to room temperature. The tubes were then capped and centrifuged at 2000 rpm for 15 min. 1 ml of the supernatant obtained from the centrifuge was transferred to other tubes. 75 *µ*l EDTA and 25 *µ*l TBA were added to 1 ml of the filtrates. The tubes were mixed in the vortex and kept in a hot water bath for 15 minutes (70°C). Then, they were brought to room temperature, and their absorbance was read on UV/Vis spectrophotometer at 532 nm [17]. *C* = *F* ∗ 6.41 ∗ *A*.

 
*C*: concentration
 
*F*: dilution factor
 
*A*: absorbance

### 2.3. Determination of SOD Activity

Superoxide dismutase (SOD) was measured with the method of “Marklund et al.” spectrophotometrically.

### 2.4. Measurement of GSH Level

800 *µ*l of phosphate buffer was added to 200 *µ*l of serum. The first absorbance (OD1) at 412 nm was recorded. 100 *µ*l of Ellman's reagent was added to the same tube, and the second absorbance was read. (OD2) was recorded. Activity (mg/ml) = [(OD2 − OD1)/13600 × E1 1.25] × 1000.

  OD1 = First absorbance before the addition of DTNB at 412 nm
  OD2 = Second absorbance after the addition of DTNB at 412 n.
  E1 = 1 in the calculations

### 2.5. Determination of GPx Activity

Serum GPx activity is based on the reduction of NADPH at 340 nm.

### 2.6. Determination of CAT Activity

Serum (0.1 ml) was added to a quartz cuvette containing 1.4 ml of 30 mM H_2_O_2_ prepared in potassium phosphate buffer (50 mM, pH 7.4). The change in absorbance was monitored by spectrophotometer at 240 nm for 30 seconds (Aeibi, 1948). Activity = (2.3/∆*x*) × [(log A1/log A2)].

  Activity: calculated in U/L
  Δ*x* = 30 seconds
  2.3 = 1 *μ*mol optical density of H_2_O_2_ in 1 cm light path

### 2.7. Statistical Analysis

Mean and standard deviation were used in the descriptive statistics of the data. One-way analysis of variance (ANOVA) was used in case of normal distribution condition in group comparisons for continuous variables. Kruskal Wallis test was used in cases where normal distribution condition was not provided. To determine the relationship between continuous variables, the Pearson correlation coefficient was calculated in cases where normal distribution condition was provided between the groups, and Spearman's rank correlation coefficient was calculated in cases where normal distribution condition was not provided. In addition, the ROC curve analysis was performed to evaluate the performance of the patient group in distinguishing it from the control group. Statistical significance level was taken as *p* < 0.05 in the calculations, and SPSS (ver: 13) package program was used for the calculations.

## 3. Results

Demographic and basic clinical features in the study population are shown in Table 1. The mean age was 23.68 ± 2.21 for subjects in the control group, 24.12 ± 3.25 for the nonobese PCOS group, and 23.11 ± 2.88 for the obese PCOS group. No statistically different results were observed between all three groups with respect to age (*p* > 0.001). There was a statistically significant difference between the study groups in the waist-hip ratio (cm), weight(kg), height (cm), BMI (kg/m^2^), menstrual cycle (day), Ferriman–Gallwey score, systolic blood pressure (SBP), and diastolic blood pressure (DBP) parameters (*p* < 0.001).

The results of the biochemical and hormone analyses are shown in Table 2. Androstenedione, total testosterone, DHEA-S, LH levels, insulin, SHBG, HDL, LDL, triglyceride, total cholesterol, and FPG levels were significantly different in the obese PCOS group compared to the nonobese PCOS and control group (*p* < 0.001) (Table 2).

However, FSH, TSH, and prolactin levels did not differ between the study groups. The mean serum MDA level was 0.273 ± 0.011 (umol/L) in the obese PCOS group, 0.229 ± 0.055 (umol/L) in the nonobese PCOS group, and 0.104 ± 0.011 (umol/L) in the healthy control group. There was a statistically significant difference in the mean serum MDA level in the obese PCOS group compared to the nonobese group and the control group (*p* < 0.001). When the nonobese PCOS group and the control group were compared in terms of the MDA value, the difference between the groups was statistically significant and high (*p* < 0.001).

When the antioxidant parameters, such as SOD, GPx, GSH, and CAT were compared with healthy the control group, nonobese PCOS group, and obese PCOS group, the difference between the groups was statistically significant (*p* < 0.001). When the antioxidant parameters were examined, it was found that these levels were significantly decreased in the obese PCOS group compared to the nonobese group (*p* < 0.001) (Table 2).

The correlation among serum MDA, GSH, GPx, SOD, and CAT levels and the clinical, biochemical, and hormonal analyses of women with PCOS are shown in Table 3.

A positive correlation was observed between MDA and BMI, triglyceride, LDL, waist-hip ratio, total cholesterol, and insulin in the PCOS patıent group. However, negatıve correlatıon was observed between SBP, DBP, SOD, CAT, GSH, and GPx. In the study, MDA's positive correlation with parameters related to metabolic syndromes, such as dyslipidemia, hypertension, and hyperinsulinemia, suggests that increased oxidative stress in PCOS patients is associated with oxidative stress in cardiovascular diseases, such as cardiovascular disease and metabolic syndrome (MS). There is a negative correlation between GSH, GPx, CAT, SOD, waist-hip ratio, and total cholesterol. There is a positive correlation between GSH, GPx, CAT, SOD, and HDL.

**Figure 1:**
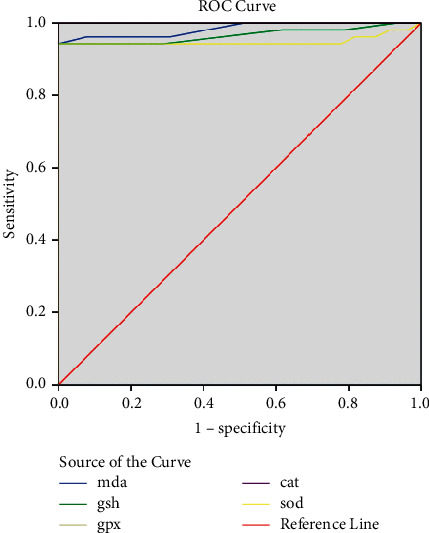
Roc curve MDA, SOD, GSH, GPx, and CAT.

Roc curve MDA, SOD, GSH, GPx, and CAT are shown in Figure 1.

In the ROC analysis, the area under the curve for MDA was 98.4%, and the cut-off point was 179.965 (confidence interval 0.961–1.0, sensitivity 0.942, and specificity 0.942) (*p* < 0.0001). The area under the curve for GSH was 96.6%, and the cut-off point was 0.13 (confidence interval 0.926–1.0, sensitivity 0.942, and specificity 0.942) (*p* < 0.0001). The area under the curve for GPx was 100%, and the cut-off point was 0.229 (confidence interval 0.1–1.0, sensitivity 1, and specificity 1) (*p* < 0.0001). The area under the curve for CAT was 100%, and the cut-off point was 328.23 (confidence interval 0.1–1.0, sensitivity 1, and specificity 1) (*p* < 0.0001). The area under the curve for SOD was 94.9%, and the cut-off point was 111.00 (confidence interval 0.892–1.0, sensitivity 0.942, and specificity 0.942) (*p* < 0.0001) (Figure 1).

## 4. Discussion

PCOS has been associated with metabolic disorders, including insulin resistance, obesity, and diabetes. Obesity has a negative impact on female fertility and is associated with anovulation, miscarriage, and several pregnancy complications. Abdominal obesity is related to insulin resistance, chronic anovulation, hyperandrogenism, and inflammation in patients with PCOS [19]. Mitochondria may be the main organ that leads to impaired energy metabolism in obese patients with PCOS. Increased oxidative stress can induce obesity by promoting preadipocyte proliferation and adipocyte differentiation by increasing the size of mature adipocytes [20].

The pathogenesis of PCOS is still complex. Also, the underlying root causes of PCOS are still unknown. The causes of PCOS may include androgen excess, abdominal adiposity, insulin resistance, and obesity. Also, the role of oxidative stress in the release of these metabolic substances is important [21].

The impaired secretion of pulsatile gonadotropin-releasing hormone (GnRH), a factor responsible for PCOS, originates from the hypothalamus. GnRH causes the pituitary gland to release the follicle-stimulating hormone (FSH) and the luteinizing hormone (LH).

These two hormones are needed for the two phases of the menstrual cycle. In PCOS, without these hormones, the egg will not form. Thus, the egg cannot leave the follicle. As a result, the cycle is disrupted, resulting in primary or secondary amenorrhea, which can be of two types. [22].

As androgen rises, it converts to estrogen peripherally. Secondly, it can feedback follicle-stimulating hormone (FSH) secretion. Thus, follicle selection may disrupt the normal system for dominant follicle development [20]. Conversely, elevated estrogen levels will increase pituitary sensitivity, resulting in increased secretion of luteinizing hormone (LH), which affects follicle development and ovulation. Also, a high LH level is a pathological risk for androgen synthesis in the ovarian theca cells. It is important in the maintenance of hyperandrogenemia. This vicious circle poses more risks to the reproductive system [23].

In our study, when LH values, which play a role in stereogenesis, are examined, it is seen that there is a significant statistical difference between the three groups. In addition, we found a negative correlation between increased LH in PCOS and GSH, an antioxidant factor.

The effects of ROS on the pathogenesis of myocardial ischemia and atherosclerosis have been identified, and studies have shown a significant correlation between the lipid peroxidase products between the plasma and arterial walls of atherosclerotic patients [24].

Metabolic syndrome is a condition with components, such as obesity associated with PCOS, high LDL, decreased HDL (high-density lipoprotein), increased fasting glucose level, increased blood pressure, and increased triglyceride levels. When one looks at the general population, metabolic syndrome increases the risk of type 2 diabetes mellitus five times and the risk of cardiovascular disease by two times [25].

Metabolic syndrome develops in patients with PCOS. Also, the rate of cardiovascular disease increases because of endothelial dysfunction in the vessels, arterial stiffness, and calcifications in the coronary arteries [26].

The measurement of blood pressure (BP), evaluation of lipid status, and glucose metabolism is metabolic syndrome screening. In statistical or regression estimates, high baseline BP is an important predictor of CVD development. Also, more than 30% of women with PCOS have an elevated BP ≥ 130/85 mmHg. In a study, it has reported that the risk of ICD10 code hypertension increased threefold in Denmark with PCOS compared to controls at baseline [27]. In a study conducted in China, it has reported that a population of women with PCOS had higher levels of lipid, glucose, insulin, and HOMA-IR than women without hypertension. Also, after adjustment for BMI [28], high BP suggests metabolic risk. Our results support that BP should be measured in all women with PCOS, regardless of BMI.

In each of the cell culture systems, reactive oxygen species (ROS) can be produced continuously as the ovarian follicles are maintained in an incubator under higher O_2_ concentrations. The overproduction of ROS can affect the IVC of preantral follicles because they can act as second messengers. It can also lead to a nonspecific pore opening in the inner mitochondrial membrane, the release of cytochrome c, and the activation of caspase cascades that ultimately result in apoptosis. The overproduction of oxidative agents can cause cell damage [29]. Oxidative stress has detrimental effects on ovarian tissue. Therefore, the use of antioxidant substances can reduce the harmful effects of free radicals on reproductive tissues. Therefore, natural products like herbal extracts can be considered to be alternative treatments for reducing ovarian tissue damage caused by oxidative stress. The loss of follicular function may weaken the antioxidant defense system. As a result, oxidative stress may damage the ovarian cell [30].

The hyperinsulinemia that occurs after an oral glucose challenge may contribute to ROS-induced oxidative stress. In PCOS, oxidative stress in response to hyperglycemia may be capable of directly stimulating hyperandrogenism. The resultant oxidative stress induces a proinflammatory state that may contribute to insulin resistance and hyperandrogenism in PCOS [31]. In a human study, it has been suggested that increased oxidative stress and decreased antioxidant capacity may contribute to the increased risk of cardiovascular diseases in women with PCOS, in addition to known risk factors, such as insulin resistance, hypertension, central obesity, and dyslipidemia [32]. The results of the studies showed that higher oxygen free radical production, evidenced by increased MDA, supports oxidative stress in polycystic ovary syndrome [33].

MDA is an important product of lipid peroxidation, and its elevated values are associated with metabolic syndrome, oxidative stress, atherosclerosis, and diabetes mellitus [34]. There are studies showing that an imbalance between the oxidant and antioxidant defense systems in patients with atherosclerotic heart disease and oxidative stress with increasing MDA values can determine the severity of atherosclerosis [15].

In our study, the MDA values in the obese PCOS group were more significant than the nonobese group, and we think that this increased oxidative stress was because of obesity. In the nonobese PCOS group, it was observed that the oxidative stress was higher than the healthy group, and the antioxidant levels decreased further. A positive correlation was observed between MDA and BMI, HOMA-IR, triglyceride, LDL, and insulin in the PCOS patıent group. When the correlation study performed in the patient group was examined, it was observed that the symptoms of PCOS-associated metabolic syndrome, such as dyslipidemia, hypertension, and hyperinsulinemia, were exacerbated parallelly with increased oxidative stress. It was found that there was a significant relationship in terms of cardiovascular risk in our patient group because of this positive correlation with hypertension findings, increased endothelial dysfunction, and hyperinsulinemia detected with MDA levels.

SOD catalyzes the dismutation of the superoxide anion radical into water and hydrogen peroxide, which is detoxified by CAT activity. SOD catalyzes the dismutation of the superoxide anion radical into water and hydrogen peroxide, which is detoxified by CAT activity [35]. Kuscu and Var determined significantly higher SOD levels in the PCOS group compared to control groups in their study. In the same study, the patients with PCOS were divided into two subgroups: the first group (who have insulin resistance) and the second group (who have no insulin resistance). SOD activity has been found significantly lower in both subgroups compared to the control [36].

In another study, MDA levels were higher and SOD values were lower in the PCOS group compared to the control group. In addition, in the same study, triglyceride (TG), LDL, and waist-hip ratio were found to be more significant, and it was suggested to monitor parameters, such as MDA, SOD, TG, and LDL during the recovery period of PCOS patients [37]. As a result, high MDA levels can be seen in patients with cellular damage.

Catalase (CAT) is an antioxidant enzyme that detoxifies H_2_O_2_ into oxygen and water and prevents cellular damage from reactive oxygen species. Hereditary catalase deficiencies have been associated with an increased risk of diabetes [38]. It was reported that lipid profiles and oxidant-antioxidant status were studied in 205 patients with PCOS patients with metabolic syndrome (MS). In addition, the decrease in antioxidant capacity in PCOS with MS was associated with increased TG and LDL-C [39]. In our study, in accordance with the literature, SOD and CAT values were significantly lower in the patient groups compared to the control group. Low antioxidant levels were observed especially in the PCOS group.. We think that hyperinsulinemia, dyslipidemia, and hypertension parameters play an active role in decreasing the antioxidant levels in obesity with increasing oxidative stress. As a result, the decreased CAT activity indicates that cellular damage occurs in the patients.

GSH acts directly as a scavenger of ROS and as a substrate for GSH peroxidase to reduce hydrogen peroxide [40]. In a study, it has been found that GSH levels in patients with PCOS are lower compared to the healthy group, and insulin resistance is associated with mitochondrial function [41]. In another study with young nonobese women, the GSH level was lower in these women than in healthy controls, independent of the status of insulin resistance [42]. In the study by Savic-Radojevic et al., PCOS women showed significantly reduced GPx activity compared to the control group, and they reported that further studies are needed to investigate the mechanism of GPx as an antioxidant defense in PCOS [43]. When the literature is examined, it is seen that different results have been obtained in studies with GSH and GPx. In our study, both GSH and GPx antioxidant levels were significantly higher in the control group compared to both PCOS groups. Low antioxidant values were determined especially in the obese PCOS group. In addition, a negative correlation was found between the waist-hip ratio (cm) and these two parameters. As a result, it can be said that low GSH level causes oxidative stress in patients.

As primary therapy, lifestyle adjustment (LSM) should be considered without the addition of metformin, regardless of fertility needs [44].

One of the main causes of aggravating PCOS is obesity, abdominal fat mass, high waist circumference, and high BMI (>25). As a result of the reduction of abdominal fat, inflammation in the body decreases. Thus, ovulation status increases as androgen level decreases. As a result, metabolic functions return to normal [45].

For patients with PCOS who do not want to have children, oral contraceptive (OC) pills should be considered as first-line therapy for long-term management [46]. Also, the current contraceptive pill can always cause venous thrombosis. It may occur from norethindrone or ethinylestradiol. New generation endogenous estrogens, such as dienogest [47], 17-estradiol, estradiol valerate, and estetrol [48] reduce the risk of treatment. It ensures that the OC treatment advantages are preserved. Ovulation treatment is an effective method for patients who need to reproduce. In a recent study, it is suggested that letrozole will replace clomiphene as the first line of treatment for ovulation [49]. For resistant ovulatory disorders, two methods can be recommended. The latest ovarian hippocampal signaling pathway block theory [50] and bilateral ovarian puncture method [51] can be selected for the induction of ovulation. The combination therapy with metformin is still the best option in patients with insulin resistance or hyperinsulinemia. Also, spirolactone may be preferred to improve hair-related symptoms in patients with different PCOS symptoms [52]. To reduce androgen levels, a safe and effective method is to use a tolerant neurokinin receptor antagonist [53]. Studies on the current treatment of PCOS are ongoing. However, its pathogenesis has not been fully elucidated. Therefore, its treatment cannot be definitively made. More studies and research are needed on PCOS.

In conclusion, in our study, it was seen that MDA values were significantly increased in both PCOS groups, especially in the obese PCOS group, and antioxidant parameters were low. Oxidative stress and decreased antioxidant parameters in PCOS patients were correlated with hyperinsulinemia, hypertension, and dyslipidemia findings, and we think that this oxidative stress condition may contribute to metabolic syndrome and cardiovascular diseases in PCOS patients. Increased oxidative stress with low antioxidants and insulin resistance and the correlation observed between these parameters indicate that women with PCOS are under oxidative stress supporting the concept that oxidative stress is involved in the pathophysiology of PCOS. In addition, the HOMA-IR level has increased in obese PCOS patients because insulin resistance has increased. On the other hand, the HOMA-IR level has not changed in nonobese PCOS patients compared to the healthy control group. It is probably because of the high levels of lipid profile parameters (LDL, triglyceride, and total cholesterol) in obese PCOS groups. Therefore, oxidative stress, in addition to known features, such as dyslipidemia, central obesity, and insulin resistance, may be a contributing factor to the risk of future cardiovascular diseases in women. Also, in the future, more oxidative stress studies should be conducted on obese and nonobese PCOS patients.

## Figures and Tables

**Table 1:** Demographic and Basic clinical features of the study groups.

	Control group (*n* = 49)	Nonobese PCOS group (*n* = 45)	Obese PCOS group (*n* = 45)	*p* value
Age (year)	23.68 ± 2.2^a^	24.12 ± 3.25^a^	23.11 ± 2.88^a^	0.760
Waist-hip ratio (cm)	0.69 ± 0.05^b^	0.074 ± 0.06^b^	0.96 ± 0.02^a^	0.001
Weight (kg)	66.18 ± 6.6^b^	67.2 ± 4.3^b^	79.23 ± 3.2^a^	0.001
Height (cm)	166.11 ± 5.5^b^	164.24 ± 4.65^b^	158.2 ± 5.2^a^	0.001
BMI (kg/m^2^)	23.24 ± 2.2^b^	24.34 ± 2.45^b^	30.21 ± 1.2^a^	0.001
SBP(mmHg)	114.42 ± 12.1^b^	112.32 ± 10.21^b^	132.2 ± 8.8^a^	0.001
DBP (mmHg)	73.21 ± 8.23^b^	75.22 ± 7.77^b^	84.23 ± 4.4^a^	0.001
Menstrual cycle (day)	23.11 ± 4.2^c^	54.16 ± 16.6^b^	69.44 ± 18.7^a^	0.001
FGS	5.32 ± 1.23^c^	9.65 ± 2.23^b^	12.45 ± 3.67^a^	0.001

a: significantly different from the obese PCOS group and the nonobese PCOS group (*p* < 0.05). b: significantly different from the obese PCOS group and controls (*p* < 0.05). c: significantly different from the nonobese PCOS group and controls (*p* < 0.05). Values are expressed as mean ± SD. Statistical significance was defined as *p* < 0.05. FGS: Ferriman–Gallwey score.

**Table 2:** Descriptive statistics and comparison results (results of biochemical parameters).

	Control group (*n* = 49)	Nonobese PCOS group (*n* = 45)	Obese PCOS group (*n* = 45)	*p* value
Androstenedione (ng/ml)	1.77 ± 0.87^**c**^	4.15 ± 1.55^b^	4.89 ± 1.22^a^	0.001
Total testosteron (ng/ml))	1.41 ± 0.68^c^	4.10 ± 1.33^b^	4.53 ± 1.31^a^	0.001
Prolactin (ng/ml)	17.60 ± 3.31^a^	19.9 ± 4.6^a^	21.7 ± 6.6^a^	0.560
DHEA-S (ug/dl)	155.8 ± 24.3^c^	302.1 ± 65.2^b^	335.3 ± 40.2^a^	0.001
FSH (mu/ml	5.4 ± 1.80^a^	4.6 ± 1.01^a^	4.4 ± 1.41^a^	0.218
LH (mu/ml)	4.63 ± 1.11^c^	8.23 ± 2.08^b^	7.55 ± 2.34^a^	0.001
Insulin (U/ml)	4.05 ± 2.02^c^	5.8 ± 3.77^b^	10.64 ± 4.90^a^	0.001
TSH (uIu/dl)	1.5 ± 0.81^a^	1.72 ± 1.33^a^	1.32 ± 0.61^a^	0.765
SHBG (nmol/l)	135.2 ± 11.49^a^	64.5 ± 14.33^b^	45.6 ± 16.54^c^	0.001
HDL (mg/dl)	53.50 ± 2.62^a^	51.21 ± 4.71^b^	39.23 ± 5.41^c^	0.001
LDL (mg/dl)	82.6 ± 7.3^c^	87.1 ± 4.6^b^	126.1 ± 18.9^a^	0.001
Triglyceride (mg/dl)	68.17 ± 12.2^c^	84.80 ± 33.7^b^	176.10 ± 21.38^a^	0.001
Total cholesterol (mg/dl)	127.11 ± 10.1^c^	158.28 ± 18.4^b^	197.28 ± 22.23^a^	0.001
FPG (mg/dl)	86.21 ± 8.81^c^	94.10 ± 12.2^b^	132.33 ± 18.2^a^	0.001
MDA (umol/L)	0.104 ± 0.011^a^	0.229 ± 0,055^b^	0.273 ± 0.011^c^	0.001
SOD (U/L)	142.410 ± 12.15^a^	82.690 ± 9.87^b^	66.320 ± 10.63^c^	0.001
GPx (u/ml)	0.840 ± 0.086^a^	0.074 ± 0.039^b^	0,061 ± 0,040^c^	0.001
CAT (u/ml)	390.256 ± 39.10^a^	175.132 ± 58.68^b^	144.100 ± 58.70^c^	0.001
GSH (mg/dl)	0.167 ± 0.009^a^	0.054 ± 0.017^b^	0.035 ± 0.019^c^	0.001

a: significantly different from the obese PCOS group and the nonobese PCOS group (*p* < 0.05). b: significantly different from the obese PCOS group and controls (*p* < 0.05). c: significantly different from the nonobese PCOS group and controls (*p* < 0.05). Values are expressed as mean ± SD. Statistical significance was defined as (*p* < 0.05). FPG: fasting plasma glucose. DHEA-S: dehydroepiandrosterone sulfate.

**Table 3:** The correlation among serum MDA, GSH, GPx, SOD, and CAT levels and the clinical, biochemical, and hormonal analyses of women with PCOS.

Correlations	MDA (umol/L)	GSH (mg/dl)	GP_X_ (u/ml)	CAT (u/ml)	SOD (U/L)
Age (year)	−0.151	−0.125	0.193	−0.145	−0.134
Waist-hip ratio (cm)	0.422^*∗∗*^	−0.488^*∗∗*^	−0.593^*∗∗*^	−0.499^*∗∗*^	−0.523^*∗∗*^
TSH (uIu/dl)	0.037	0.085	0.106	−0.112	−0.011
Total cholesterol (mg/dl)	0.204^*∗*^	−0.349^*∗*^	−0.385	−0.675^*∗∗*^	−0.414^*∗∗*^
Total testosteron (ng/ml)	−0.174	−0.052	−0.193	−0.234	−0.118
Prolactin (ng/ml)	0.202	−0.194	0.189	0.167	0.136
LH (mU/ml)	0.024	−0.206^*∗∗*^	0.309	0.776	0.763
LDL (mg/dl)	0.504^*∗∗*^	−0.466	−0.179^*∗*^	−0.471^*∗*^	−0.487^*∗*^
Insulin (U/ml)	0.785^*∗∗*^	−0.079	−0.386^*∗*^	−0.727^*∗*^	−0.703^*∗*^
HDL (mg/dl)	0.357	0.414^*∗*^	0.442^*∗*^	0.483^*∗∗*^	0.524^*∗∗*^
FSH (mU/ml)	−0.001	−0.002	0.047	0.020	0.003
Triglyceride	0.786^*∗∗*^	−0.321^*∗*^	−0.125	−0.354^*∗*^	−0.417^*∗*^
SBP(mmHg)	−0.635^*∗∗*^	−0.113	−0.227	−0.381^*∗*^	−0.279^*∗*^
DBP(mmHg)	−0.695^*∗∗*^	−0.272	−0.200	−0.237	−0.138
BMI(kg/m^2^)	0.584^*∗∗*^	−0.439^*∗∗*^	−0.482^*∗*^	−0.146	−0.273^*∗∗*^
Androstenedione (ng/ml)	−0.203	0.161	0.205	0.132	0.237
MDA(umol/L)	—	−0.961^*∗∗*^	−0.914^*∗∗*^	−0.880^*∗∗*^	−0.939^*∗∗*^
GSH (mg/dl)	−0.961^*∗∗*^	—	0.893^*∗∗*^	0.854^*∗∗*^	0.913^*∗∗*^
GP_X_(u/ml)	−0.914 ^*∗∗*^	0.893^*∗∗*^	—	0.841^*∗∗*^	0.845^*∗∗*^
CAT (u/ml)	−0.880^*∗∗*^	0.854^*∗∗*^	0.841^*∗∗*^	—	0.802^*∗∗*^
SOD (U/L)	−0.939 ^*∗∗*^	0.913^*∗∗*^	0.945^*∗∗*^	0.802^*∗∗*^	—

^*∗*^ *p* < 0.05, ^*∗∗*^*p* < 0.01.

## Data Availability

The data used to support the findings of this study are included within the article.
